# High Population Frequency of *GNRHR* p.Q106R in Malta: An Evaluation of Fertility and Hormone Profiles in Heterozygotes

**DOI:** 10.1210/jendso/bvad172

**Published:** 2023-12-29

**Authors:** Clayton John Axiak, Adrian Pleven, Ritienne Attard, Francesca Borg Carbott, Jean-Paul Ebejer, Ian Brincat, Karen Cassar, Mark Gruppetta, Josanne Vassallo, Stephanie Bezzina Wettinger, Rosienne Farrugia

**Affiliations:** Department of Applied Biomedical Science, Faculty of Health Sciences, University of Malta, Msida, MSD 2080, Malta; Department of Applied Biomedical Science, Faculty of Health Sciences, University of Malta, Msida, MSD 2080, Malta; Clinical Chemistry Section, Department of Pathology, Mater Dei Hospital, Msida, MSD 2080, Malta; Department of Applied Biomedical Science, Faculty of Health Sciences, University of Malta, Msida, MSD 2080, Malta; Department of Applied Biomedical Science, Faculty of Health Sciences, University of Malta, Msida, MSD 2080, Malta; Centre for Molecular Medicine and Biobanking, University of Malta, Msida, MSD 2080, Malta; Clinical Chemistry Section, Department of Pathology, Mater Dei Hospital, Msida, MSD 2080, Malta; Department of Medicine, Faculty of Medicine and Surgery, University of Malta, Msida, MSD 2080, Malta; Department of Medicine, Faculty of Medicine and Surgery, University of Malta, Msida, MSD 2080, Malta; Division of Endocrinology and Diabetes, Department of Medicine, Mater Dei Hospital, Msida, MSD 2080, Malta; Centre for Molecular Medicine and Biobanking, University of Malta, Msida, MSD 2080, Malta; Department of Medicine, Faculty of Medicine and Surgery, University of Malta, Msida, MSD 2080, Malta; Division of Endocrinology and Diabetes, Department of Medicine, Mater Dei Hospital, Msida, MSD 2080, Malta; Department of Applied Biomedical Science, Faculty of Health Sciences, University of Malta, Msida, MSD 2080, Malta; Centre for Molecular Medicine and Biobanking, University of Malta, Msida, MSD 2080, Malta; Department of Applied Biomedical Science, Faculty of Health Sciences, University of Malta, Msida, MSD 2080, Malta; Centre for Molecular Medicine and Biobanking, University of Malta, Msida, MSD 2080, Malta

**Keywords:** idiopathic hypogonadotropic hypogonadism, infertility, puberty, GnRH deficiency, *GNRHR* founder effect, hormone profiles

## Abstract

**Context:**

The gonadotropin-releasing hormone receptor variant *GNRHR* p.Q106R (rs104893836) in homozygosity, compound heterozygosity, or single heterozygosity is often reported as the causative variant in idiopathic hypogonadotropic hypogonadism (IHH) patients with GnRH deficiency. Genotyping of a Maltese newborn cord-blood collection yielded a minor allele frequency (MAF) 10 times higher (MAF = 0.029; n = 493) than that of the global population (MAF = 0.003).

**Objective:**

To determine whether *GNRHR* p.Q106R in heterozygosity influences profiles of endogenous hormones belonging to the hypothalamic-pituitary axis and the onset of puberty and fertility in adult men (n = 739) and women (n = 239).

**Design, Setting, and Participants:**

Analysis of questionnaire data relating to puberty and fertility, genotyping of the *GNRHR* p.Q106R variant, and hormone profiling of a highly phenotyped Maltese adult cohort from the Maltese Acute Myocardial Infarction Study.

**Main Outcome and Results:**

Out of 978 adults, 43 *GNRHR* p.Q106R heterozygotes (26 men and 17 women) were identified. Hormone levels and fertility for all heterozygotes are within normal parameters except for TSH, which was lower in men 50 years or older.

**Conclusion:**

Hormone data and baseline fertility characteristics of *GNRHR* p.Q106R heterozygotes are comparable to those of homozygous wild-type individuals who have no reproductive problems. The heterozygous genotype alone does not impair the levels of investigated gonadotropins and sex steroid hormones or affect fertility. *GNRHR* p.Q106R heterozygotes who exhibit IHH characteristics must have at least another variant, probably in a different IHH gene, that drives pathogenicity. We also conclude that *GNRHR* p.Q106R is likely a founder variant due to its overrepresentation and prevalence in the island population of Malta.

Idiopathic hypogonadotropic hypogonadism (IHH) is a rare genetic disorder that is characterized by partial or complete absence of pubertal development. Dysfunction of the hypothalamic-pituitary-gonadal axis leads to disorders of development, sexual maturation, and reproduction. Disruption in migration, differentiation or activation of GnRH neurons and/or a disruption in production, pulsatile secretion, or action of GnRH [1-3] lead to varying degrees of gonadotropin secretion and subsequently hypogonadism as determined by low testosterone levels and absence of gametogenesis. It is classically termed Kallmann syndrome if it presents with anosmia or hyposmia, and normosmic IHH if the sense of smell is not impaired [4].

The GnRH decapeptide that is released from specialized neurons in the medio-basal and anterior hypothalamus binds to GnRH receptors located in the adenohypophyseal gonadotrope cell membranes. Once activated, these cells synthesize and release gonadotropins: FSH, and LH [5, 6]. Gonadotropin secretion stimulates gametogenesis and gonadal steroidogenesis [7, 8]. However, pathogenic variation in the GnRH receptor (*GNRHR*; OMIM 138850; phenotype MIM 146110; GRCh38 genomic coordinates: 4:67 737,117–67 754 387) may compromise the interaction with GnRH and result in IHH [8-11]. GnRH or GnRH receptor variants may also lead to abnormalities of downstream signalling such as intracellular calcium ion fluxes, cAMP signalling, inositol triphosphate generation, DNA transcription, synthesis, and secretion of gonadotropins [7, 12].

The GnRH receptor is a 328 amino acid long polypeptide transmembrane rhodopsin-like G protein-coupled receptor. *GNRHR* was among the first genes to be implicated in IHH, and it is classically associated with a recessive mode of inheritance, where heterozygous individuals are generally asymptomatic [13-15]. Most reported variants with a suspected functional effect in *GNRHR* are missense changes that impede GnRH signaling. There is an increasing amount of data suggesting that heterozygous variants may lead to diminished receptor signaling through various mechanisms. These include the loss or reduction of receptor expression due to the rerouting of misfolded proteins toward degradation pathways instead of localization to the cell membrane, a decrease in ligand binding because of variants in the ligand binding pocket, or a decrease in G-protein coupling or signalling due to variants in the signaling domains [9, 15, 16]. It has been demonstrated that the prevalence of heterozygous *GNRHR* variants is statistically significantly higher in individuals with GnRH deficiency when compared with controls (2.5% in cases, 0.5% in controls, *P* < .01), suggesting that monoallelic expression might impact reproductive function possibly when combined with other genetic or environmental factors [15]. While dominant-negative effects of monoallelic *GNRHR* variants have been previously described *in vitro*, one cannot ignore the possibility that *in vivo* either oligogenicity or nongenetic factors may also play a role, and that additional genes that play a role in IHH are still to be determined [17, 18].

The most frequently reported pathogenic *GNRHR* variant across different ethnic groups is p.Q106R (rs104893836) [10, 19, 20]. This glutamine to arginine substitution at residue 106 sits on the first extracellular hydrophobic loop of the G protein-coupled receptor [21]. The variant causes a conformational change in the receptor and diminishes ligand binding and receptor activation, resulting in partial loss-of-function [10, 13, 22]. *In vitro* expression data shows that the p.Q106R variant receptor needs 50 times higher levels of the ligand for half-maximal production of the secondary messenger inositol phosphate (IP3) [23]. Additionally, this variant also compromises cAMP signaling pathways and the activation of extracellular signal-regulated kinase (ERK) [24, 25]. Downstream to these pathways, the stimulation of the pituitary gonadotropin alpha-subunit, LH-beta, and FSH-beta gene promoter activity and expression are also reduced when compared to the wild-type [25].

Here we report an atypically high frequency of *GNRHR* p.Q106R in the Maltese population, which presented an opportunity to conduct an endocrine assessment of heterozygous individuals between 18 and 77 years old. We also investigate reported puberty and fertility parameters in the identified *GNRHR* p.Q106R heterozygotes.

## Materials and Methods

### Research Subjects

Blood samples and clinical, biological, and questionnaire data were collected after obtaining written informed consent. Genetic data for this project originated from 3 independent cohorts: (1) the Malta Next Generation Sequencing (NGS) Project (ethics approval: 031/2014) that uses high throughput sequencing (HTS) to investigate selected conditions in the Maltese (n = 146; 17 of whom form part of the IHH cohort, diagnosed [26] and recruited through the Reproductive Endocrinology Clinic at Mater Dei Hospital, Msida, Malta); (2) the Maltese Acute Myocardial Infarction (MAMI) study [27], which includes individuals between the ages of 18 and 77 years and for which extensive phenotypic and biochemical data, including hormone levels, are available (n = 1098; ethics approval: 32/2010). Of these, 423 are cases with a first myocardial infarction (MI) recruited at time of MI and recalled again at least 6 months post first MI, 210 are relatives of the cases, and 465 are population controls; and (3) an anonymous cord blood population cohort of Maltese neonates collected over a period of 3 months using a convenience consecutive sampling approach (n = 493; ethics approval: 44/2014). For the NGS project and the MAMI study samples, medical, demographic, and lifestyle data was collected through an extensive interviewer-led questionnaire. Serum and plasma samples were collected at time of recruitment and recoded prior to testing. All research subjects for the NGS project and the MAMI study have parents and grandparents of Maltese ethnicity.

### DNA Analysis

DNA was extracted from EDTA whole blood or buffy coats using the salting out method [28]. Several methods were employed to genotype the *GNRHR* variant (NM_000406.2: c.317A > G; P30968: p.Q106R) in the different sample cohorts.

For the Malta NGS project, a custom targeted panel HTS approach that selectively captures all exons and up to 500 bp of flanking regions of the gene was used. *GNRHR* was among the selected genes on this panel. DNA libraries were constructed according to the Agilent SureSelect^XT^ Target Enrichment System for Illumina Paired-End Sequencing Library Protocol Version B.3. Libraries were subsequently sequenced on an Illumina Hi-Seq 4000 platform at BGI, Hong Kong. HTS data was aligned to GRCh37 and variants called using NextGENe v.2.4.2.3 (SoftGenetics, LLC). Variants were filtered on a minimum of 30-fold coverage.

A restriction enzyme digest with *Xcml* (NEB, UK) was used to genotype the neonatal cord blood collection [29]. The enzyme cleaves the wild-type but not the alternative allele. Restriction fragments were separated on 2% agarose gel and genotypes confirmed by Sanger sequencing.

For the MAMI study collection, the Kompetitive Allele Specific PCR (KASP™) genotyping assay for *GNRHR* p.Q106R was carried out at LGC Genomics, Germany. This assay is based on competitive allele-specific PCR that allows for bi-allelic scoring of the single nucleotide variant. This is quantified on the basis of fluorescence resonant energy transfer chemistry [30]. The controls were tested for Hardy–Weinberg equilibrium using Fisher's exact test, and the genotype and allele frequencies were calculated. Genotypes were confirmed by Sanger sequencing and by comparison to HTS datasets.

### Hormone Assays and Fertility Data

Hormone assays were carried out on serum samples from the MAMI study collection. To limit diurnal variation, blood from overnight fasting subjects was always drawn in the morning between 0800 hours and 1000 hours. Samples were processed, aliquoted, and stored at −80 °C within 90 minutes of collection. At time of measurement, the frozen aliquots were utilized immediately after thawing. Samples were only thawed once.

Diagnostic enzyme-amplified chemiluminescent immunoassays (Immulite 2000 System, Siemens, USA) were carried out on all samples in the MAMI study collection to determine the concentration of several hormones that have a role in the hypothalamic-pituitary axis. Instrument calibration was carried out weekly for testosterone and every 4 weeks for the remaining hormones. Before each run, internal quality control checks were carried out. The hormones tested included estradiol (women only; Siemens Cat# L2KE22, RRID: AB_2936944), total testosterone (men only; Siemens Cat# L2KTW2, RRID: AB_2756391), lutenising hormone (LH; Siemens Cat# L2KLH2, RRID: AB_2756388), follicle stimulating hormone (FSH; Siemens Cat# L2KFS2, RRID: AB_2756389), sex hormone-binding globulin (SHBG; Siemens Cat# L2KSH2, RRID: AB_2819251), dehydroepiandrosterone sulfate (DHEA-SO_4_; Siemens Cat# L2KDS2, RRID: AB_2895591), prolactin (Siemens Cat# L2KPR2, RRID: AB_2827375), cortisol (Siemens Cat# LKCO2, RRID: AB_2810257), thyroid stimulating-hormone (TSH; Siemens Cat# LKTS1, RRID: AB_2827386), free T4 (Siemens Cat# LKFT41, RRID: AB_2827385), and growth hormone (GH; Siemens Cat# L2KGRH2, RRID: AB_2811291). All quantitative measurements were carried out according to the manufacturer's instructions. Hormone measurements that fell outside the detection limit of the kit were subsequently omitted from the analysis. The exception was estradiol, where nondetectable values (denoted ND) are given as the lower limit of the range and considered to be part of the reference range by the assay. Calculated free testosterone was computed from total testosterone and SHBG using the Vermeulen equation [31]. Since albumin measurements were not available, a standard value of 43 g/L was used.

Age, sex, and data associated with puberty and fertility including age of menarche and frequency of menstruation and need and use of fertility treatment in women, number of offspring, and marital status were obtained from an interviewer-led questionnaire. We did not assess puberty and fertility in men through direct questions but used family tree information to determine number of offspring. Individuals who previously had cancer, an oophorectomy, or hysterectomy at pre- or perimenopausal age and those on hormone replacement therapy were omitted from this analysis, resulting in 978 samples: 739 men and 239 women for whom demographic, genetic, and hormone data were available.

### Statistical Data Analysis

Statistical analyses were conducted using SPSS Statistics version 25 (IBM Corp. Armonk, NY, USA). The neonatal collection and MAMI control group were tested for the proportion of genotypes and deviation from the Hardy–Weinberg equilibrium using Fisher's exact test. Nonparametric tests were used for hormone analysis due to the nonnormal distribution of the data. The Kruskal–Wallis test was used to compare 3 or more independent samples of unequal sample sizes, and the Mann–Whitney test was used to carry out pair-wise comparisons. The α threshold employed was arbitrarily set at *P* < .05.

Prior to hormone analysis, medians and *P*-values were calculated for all hormones by sex, control-case-relative status, and 10-year age groups to identify whether sex, age, or case-control-relative status influenced hormone levels. Median hormone levels were only found to be statistically different between men and women. An age trend was also observed in DHEA-SO_4,_ GH, SHBG, and free testosterone in men and DHEA-SO_4_, estradiol, FSH, and LH in women. Thus, data is presented separately for men and women and subdivided into <50 and ≥50 years of age. For DHEA-SO_4_, SHBG, estradiol, LH, and FSH in women, data is subdivided into pre-/perimenopausal and postmenopausal status.

Scatter plots for hormone levels by genotype across the different age groups were constructed using GraphPad Prism version 8.0.2 (GraphPad Software Inc, San Diego, CA, USA).

## Results

Analysis of HTS data of 146 research subjects from the Malta NGS project identified 8 heterozygotes harboring the *GNRHR* p.Q106R variant. Four of these (2 of whom were parent and offspring) formed part of the IHH cohort (n = 17), while the other 4 did not have any IHH characteristics, nor a family history of IHH. A local population study on a cord blood DNA collection (n = 493) representative of the current Maltese population identified 2 homozygous alternative and 25 heterozygous individuals, all unrelated, translating to a population MAF of 0.029 [29]. This is considerably higher than the MAFs of the European population or its subpopulations, with the lowest reported variant frequency being 0.0012 in the Estonian population and the highest being 0.0051 in the southern European population [32]. For a variant that is reported to be pathogenic in multiple literature accounts, its frequency is overrepresented in the Maltese population, and this led us to evaluate the effect of *GNRHR* p.Q106R heterozygosity on fertility and hormone profiles in an adult cohort.

### Clinical and Demographic Characteristics

**Figure 1.**
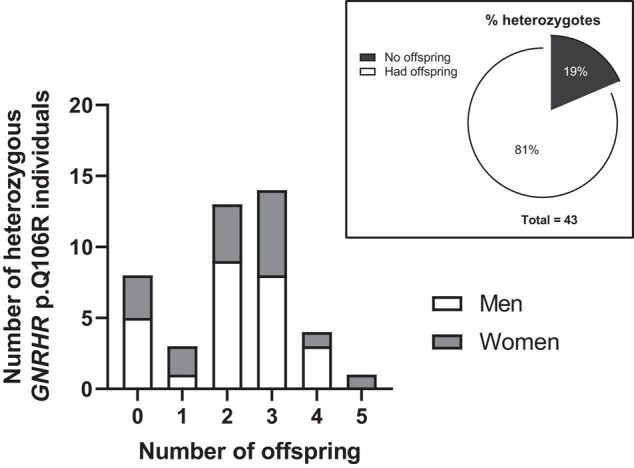
Frequency and percentage distribution of offspring by sex for individuals that are heterozygous for *GNRHR* p.Q106R. Of the 8 without offspring, 6 are single and have never been in a steady relationship.

From 978 eligible individuals of the MAMI study collection, 43 (4.4%) were heterozygous for *GNRHR* p.Q106R (Fig. 1). These consisted of 26 men with ages ranging from 27 to 68 years and 17 women between the age of 23 and 75 years. The genetic variant was in the Hardy–Weinberg equilibrium in controls with MAF = 0.033. From the 43 heterozygotes, 35 had offspring. In heterozygous women (Table 1), menarche occurred between the ages of 9 and 14 years. Menstrual bleeding lasted 3 to 8 days, and the number of days elapsed between each cycle ranged from 25 to 34 days; only 1 woman required fertility treatment. Of the 8 heterozygotes without offspring (3 women, ages 23, 23, and 27 years, and 5 men, ages 27, 31, 53, 59, and 68 years), only 2 men aged 31 and 68 years are, or have been, in a steady relationship.

**Table 1.** Demographic, puberty, and fertility characteristics of women

	Women wild-type for *GNRHR p.Q106R* (n = 281)	Women heterozygous for *GNRHR p.Q106R* (n = 17)
Mean age ± SD in years (range)	45.1 ± 15.0 (18–77)	53.4 ± 17.9 (23–75)
Mean age at menarche ± SD in years (range)	12.6 ± 1.6 (8–17)	11.4 ± 1.7 (9–14)
Median days of menstrual flow (range)	5 (2–10)	5 (3–8)
Median number of days between menstrual cycles (range)	28 (15–100)	28 (25–34)
Menstrual regularity	86.8% regular, 13.2% irregular	88.2% regular, 11.8% irregular
Fertility treatment required	4.4% yes, 95.6% no	5.9% yes, 94.1% no
Median number of offspring; including miscarriages (range)	2 (0–7)	2 (0–5)
Median BMI; kg/m^2^ (range)	28.3 (16.2–60.5)	26.4 (20.6–38.6)
Underweight (BMI < 18.5) (%)	1.5	0
Healthy range (18.5 ≤ BMI ≤ 24.9) (%)	27.1	35.3
Overweight (25 ≤ BMI ≤ 29.9) (%)	34.2	29.4
Obese (30 ≤ BMI ≤ 39.9) (%)	33.1	35.3
Severely obese (BMI ≥ 40) (%)	4.1	0

Abbreviations: BMI, body mass index.

### Hormone Assays

Upon analysis of research subjects grouped into 10-year age groups (data not shown), we observed a steady decline in DHEA-SO_4_ levels with age in both sexes, an increase in SHBG levels with age in men, an age-dependent decrease in free testosterone in men and estradiol in women, and an overall age-dependent FSH and LH increase in women up until menopause. These trends are reflective of the natural biological aging process [33-35].

**Figure 2.**
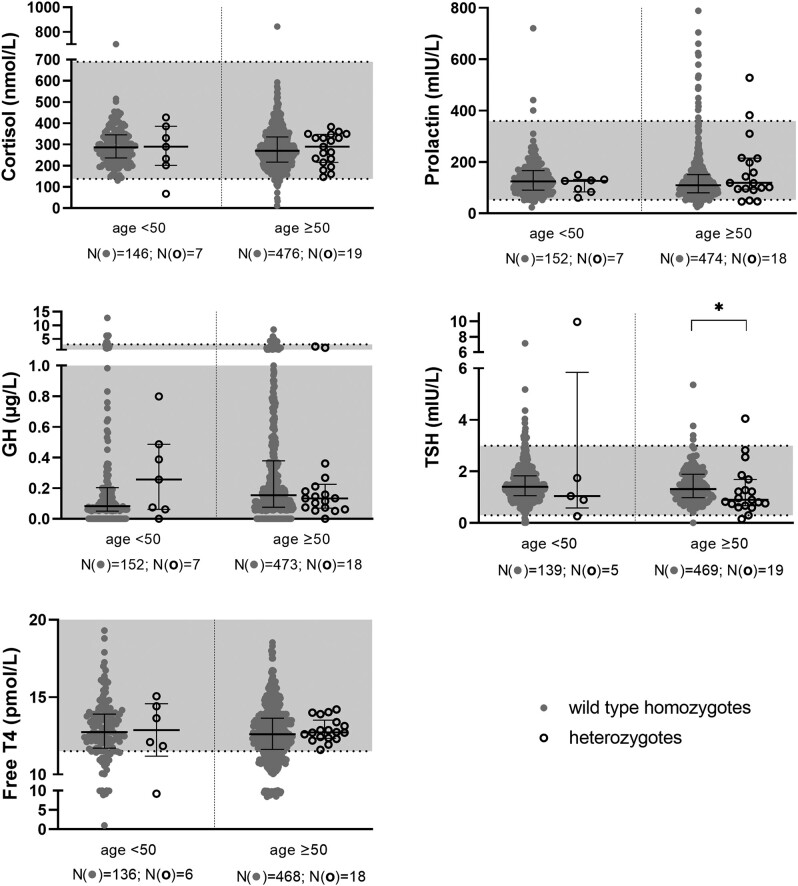
Scattergrams (median and interquartile ranges) by zygosity in men with age <50 and ≥50 years in the Maltese Acute Myocardial Infarction cohort for cortisol, prolactin, GH, TSH, and free T4. Median levels between the genotypes were compared using the Kruskal–Wallis test. Solid circles are used to show wild-type samples and open circles show heterozygous samples. Data points that fall within the grey area along the y-axis are within the reference range of each respective hormone. There is no statistically significant difference between the median hormone levels of heterozygous and wild-type individuals unless marked. * *P* < .01.

TSH levels were lower in heterozygous men older than 50 years of age compared to levels in homozygous wild-type men (*P* < .01; Fig. 2, Table 2). A similar trend was also observed in men below age 50; however, this did not reach statistical significance possibly due to the small number of heterozygotes. In women the difference was much smaller and did not reach statistical significance in any of the age groups. There were no statistically significant differences in median levels between wild-type individuals and heterozygotes for prolactin, GH, DHEA-SO_4_, cortisol, SHBG, free T4, LH, and FSH in both sexes, estradiol in women, and free testosterone in men. The only statistical difference (*P* < .01) was for TSH in men above age 50 where wild-type individuals had higher levels than heterozygotes.

**Table 2.** Median hormone levels for *GNRHR* p.Q106R heterozygotes vs wild-type individuals

		Men				Women		
Hormone	Age group	Wild-type Median (n)	Heterozygotes Median (n)	*P*-value	Age group	Wild-type Median (n)	Heterozygotes Median (n)	*P*-value
Estradiol (pmol/L)	<50 years	—	—	—	Pre/perimenopausal	217.00 (94)	260.50 (6)	.75
Free testosterone (nmol/L)		0.30 (147)	0.29 (7)	0.90		—	—	—
SHBG (nmol/L)		29.20 (153)	28.20 (7)	0.24		65.30 (86)	57.25 (6)	.59
DHEA-SO_4_ (µmol/L)		5.51 (151)	7.49 (7)	0.39		3.31 (94)	4.89 (6)	.09
LH (IU/L)		3.66 (145)	4.22 (6)	0.30		5.38 (91)	5.29 (4)	.99
FSH (IU/L)		3.50 (145)	4.98 (6)	0.25		5.31 (92)	5.81 (4)	.85
Cortisol (nmol/L)		286.94 (146)	289.70 (7)	0.83	<50 years	238.88 (82)	243.50 (6)	.78
Prolactin (mIU/L)		124.00 (152)	126.00 (7)	0.40		177.0 (92)	222.50 (6)	.78
GH (ng/mL)		0.08 (152)	0.26 (7)	0.26		0.39 (92)	0.41 (6)	.67
TSH (mIU/L)		1.33 (139)	1.05 (5)	0.64		1.38 (76)	1.26 (6)	.26
Free T4 (pmol/L)		12.74 (136)	12.87 (6)	0.99		12.69 (78)	13.64 (6)	.10
Estradiol (pmol/L)	≥50 years	—	—	—	Postmenopausal	0.00 (164)	0.00 (10)	.67
Free testosterone (nmol/L)		0.24 (451)	0.28 (18)	0.94		—	—	—
SHBG (nmol/L)		42.25 (477)	31.60 (19)	0.53		52.50 (167)	55.10 (10)	.77
DHEA-SO_4_ (µmol/L)		2.81 (473)	3.04 (17)	0.73		1.69 (166)	1.15 (10)	.17
LH (IU/L)		4.63 (469)	4.05 (19)	0.07		25.10 (164)	28.50 (10)	.75
FSH (IU/L)		5.59 (470)	5.32 (19)	0.39		61.50 (164)	52.20 (10)	.56
Cortisol (nmol/L)		269.83 (476)	290.00 (19)	0.92	≥50 years	252.36 (166)	224.31 (10)	.59
Prolactin (mIU/L)		108.00 (474)	118.50 (18)	0.29		112.00 (172)	85.00 (10)	.23
GH (ng/mL)		0.15 (473)	0.13 (18)	0.55		0.18 (172)	0.22 (10)	.26
TSH (mIU/L)		1.42 (469)	0.91 (19)	**0**.**01**		1.59 (154)	1.36 (10)	.63
Free T4 (pmol/L)		12.60 (468)	12.72 (18)	0.39		13.00 (163)	11.80 (10)	.07

Abbreviations: DHEA-SO_4_, dehydroepiandrosterone sulfate; SHBG, sex hormone-binding globulin.

Statistically significant *P*-values are highlighted in bold. n, number of individuals in the group.

**Figure 3.**
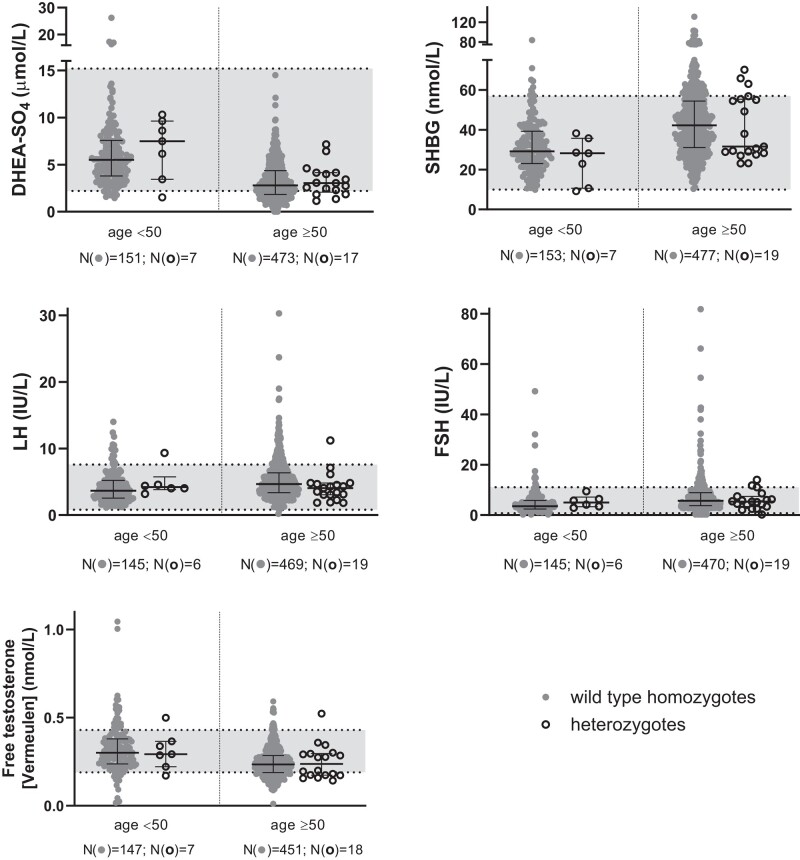
Scattergrams (median and interquartile ranges) by zygosity in men with age <50 and ≥50 years in the Maltese Acute Myocardial Infarction cohort for dehydroepiandrosterone sulfate (DHEA-SO_4_), sex hormone-binding globulin (SHBG), LH, FSH, and calculated free testosterone. Median levels between the genotypes were compared using the Kruskal–Wallis test. Solid circles are used to show wild-type samples and open circles show heterozygous samples. Data points that fall within the grey area along the y-axis are within the reference range of each respective hormone. There is no statistically significant difference between the median hormone levels of heterozygous and wild-type individuals.

**Figure 4.**
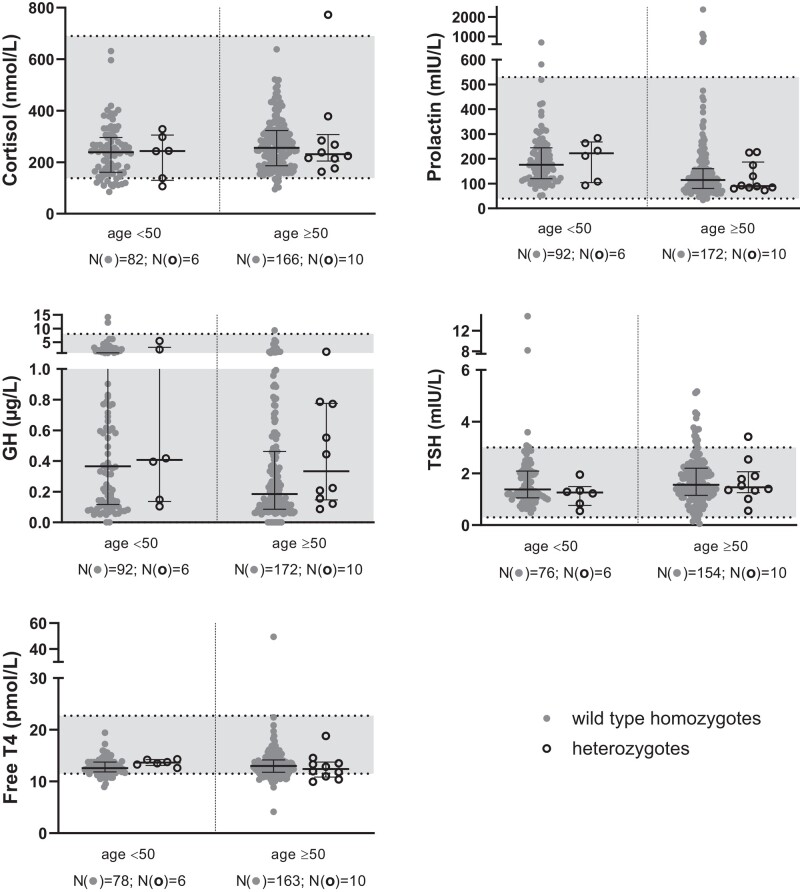
Scattergrams (median and interquartile ranges) by zygosity in women with age <50 and ≥50 years in the Maltese Acute Myocardial Infarction cohort for cortisol, prolactin, GH, TSH, and free T4. Median levels between the genotypes were compared using the Kruskal–Wallis test. Solid circles are used to show wild-type samples and open circles show heterozygous samples. Data points that fall within the grey area along the y-axis are within the reference range of each respective hormone. There is no statistically significant difference between the median hormone levels of heterozygous and wild-type individuals.

**Figure 5.**
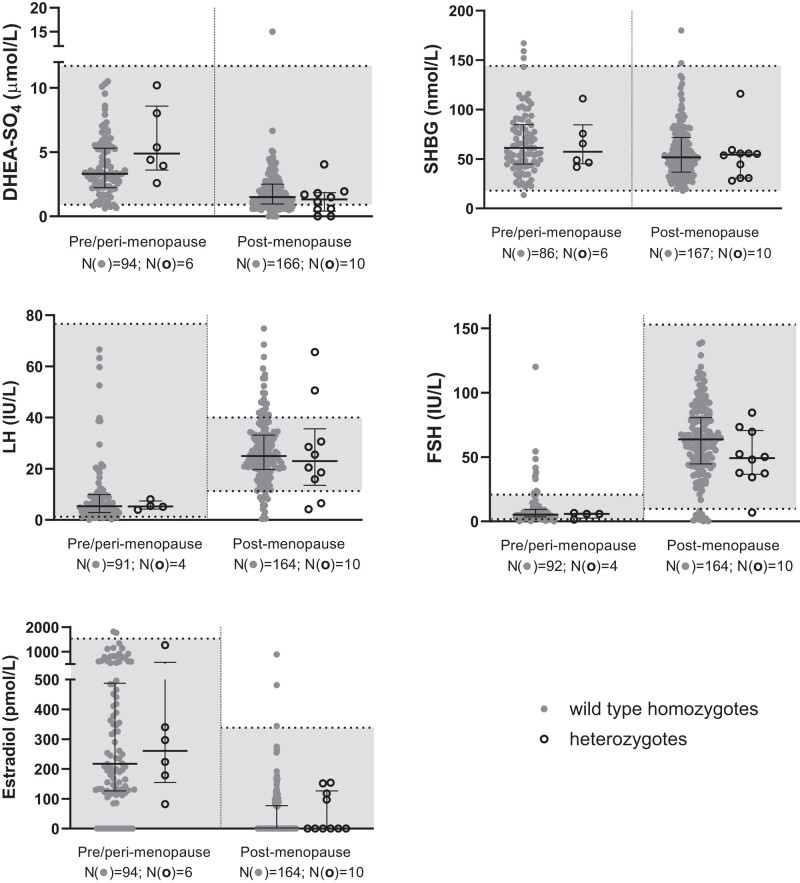
Scattergrams (median and interquartile ranges) by zygosity in women categorized by pre/perimenopausal and postmenopausal status in the Maltese Acute Myocardial Infarction cohort for dehydroepiandrosterone sulfate (DHEA-SO_4_), sex hormone-binding globulin (SHBG), LH, FSH, and estradiol. Median levels between the genotypes were compared using the Kruskal–Wallis test. Solid circles are used to show wild-type samples and open circles show heterozygous samples. Data points that fall within the grey area along the y-axis are within the reference range of each respective hormone. There is no statistically significant difference between the median hormone levels of heterozygous and wild-type individuals.

The 43 *GNRHR* p.Q106R heterozygotes in the collection were spread across different age groups with a comparable proportion of heterozygotes to wild-type homozygotes in each age group for both sexes. Across the different hormones, there were no notable differences or trends between the measured hormone levels of both genotypes and the reference ranges as most data points fell within the local hospital reference ranges (Figs. 2–5, Table 3).

**Table 3.** **Baseline characteristics and hormone levels of *GNRHR* p**.**Q106R heterozygotes**

Research subject	Age	Sex	Estradiol (pmol/L)	LH (IU/L)	FSH (IU/L)	Calculated free testosterone (nmol/L)	SHBG (nmol/L)	DHEA-SO_4_ (µmol/L)	Cortisol (nmol/L)	Free T4 (pmol/L)	TSH (mIU/L)	Prolactin (mIU/L)	GH (ng/mL)	Number of children
			Reference ranges											
			ND—1468 (pre-F) ND—374 (post-F) ND—206 (M)	1.1–77 (pre-F) 11.3–39.8 (post-F) 0.8–7.6 (M)	1.2–21 (pre-F) 21.7–153 (post-F) 0.7–11.1 (M)	0.19–0.43 (M)	18–144 (F) 10–57 (M)	0.9–11.7 (F) 2.2–15.2 (M)	138–690	12–30	0.3–3	40–530 (F) 53–360 (M)	≤8 (F) ≤3 (M)	
1	23	*^a^* F	179.0	8.1	5.8	—	65.6	3.9	298.0	13.3	1.3	108.0	5.5	0
2	23	*^a^* F	1270.0	4.0	1.5	—	46.4	8.0	243.0	14.2	2.0	92.9	0.4	0
3	27	*^a^* F	81.9	5.1	6.5	—	111.0	10.2	244.0	14.3	1.2	263.0	0.1	0
4	34	*^a^* F	224.0	—	—	—	75.6	2.6	328.3	13.5	0.8	233.0	2.4	3
5	44	*^a^* F	340.0	—	—	—	48.9	4.4	**137**.**7**	13.8	0.5	284.0	0.1	1
6	44	*^a^* F	297.0	5.5	5.8	—	41.8	5.4	106.2	12.6	1.3	212.0	0.4	2
7	55	F	118.0	65.6	73.3	—	55.1	**0**.**5**	163.3	**10**.**0**	1.8	92.4	0.8	3
8*^b^*	55	F	154.0	4.2	6.9	—	59.2	1.7	176.3	13.5	1.4	227.0	0.1	3
9	57	F	ND	50.6	52.2	—	30.8	1.2	238.0	13.3	1.9	175.0	0.1	2
10	61	F	*—*	—	—	—	*—*	*—*	*—*	*—*	*—*	*—*	*—*	2
11*^b^*	62	F	97.3	25.5	37.7	—	30.7	**0**.**6**	267.6	12.8	2.5	225.0	0.4	3
12	63	F	ND	28.5	37.3	—	44.5	1.9	284.2	14.5	1.3	88.6	0.2	5
13	66	F	152.0	15.9	34.3	—	28.0	ND	216.3	12.0	0.6	84.0	0.8	1
14*^b^*	72	F	ND	20.5	50.1	—	53.7	1.8	**772**.**5**	18.8	1.5	130.0	1.5	2
15	73	F	ND	18.6	69.6	—	116.0	4.0	214.0	**11**.**8**	**3**.**4**	79.7	0.6	3
16	74	F	ND	6.5	48.1	—	55.5	1.5	224.3	**11**.**0**	1.4	73.1	0.2	3
17	75	F	ND	30.6	84.4	—	55.5	ND	378.0	**10**.**4**	1.0	85.0	0.2	4
18	27	M	*—*	9.3	9.5	**0**.**50**	10.7	10.3	427.7	14.4	0.9	150.0	0.4	0
19	28	M	*—*	—	—	0.29	22.9	7.5	201.7	15.1	1.1	126.0	0.5	3
20	31	M	*—*	3.2	2.9	0.37	28.2	6.2	289.7	13.6	*—*	61.1	0.3	0
21	39	M	*—*	4.6	3.4	**0**.**17**	38.2	3.5	233.1	12.1	0.3	95.0	0.1	2
22	47	M	*—*	4.4	6.4	0.29	35.7	**1**.**5**	**68**.**2**	**11**.**8**	1.8	128.0	0.1	2
23	49	M	*—*	4.0	5.8	0.22	**9**.**2**	9.6	331.1	*—*	*—*	83.7	ND	2
24	49	M	*—*	4.0	4.2	0.34	28.6	8.6	386.0	**9**.**2**	**9**.**9**	132.0	0.8	2
25	50	M	*—*	4.3	4.5	0.28	27.0	6.4	234.0	13.4	0.9	**46**.**9**	0.1	3
26	51	M	*—*	3.4	5.8	**0**.**16**	28.3	7.2	160.9	12.9	1.3	143.0	0.2	2
27	52	M	*—*	1.9	0.2	0.35	55.0	4.2	147.0	12.7	1.7	382.0	0.2	2
28	53	M	*—*	1.9	4.2	**0**.**18**	29.1	4.6	179.1	12.9	0.3	118.0	ND	3
29	53	M	*—*	4.3	6.2	0.36	23.1	4.2	350.4	12.4	0.8	198.0	0.1	0
30	54	M	*—*	1.8	2.5	0.29	49.2	4.0	383.5	13.9	0.6	214.0	2.2	2
31	55	M	*—*	3.1	6.0	—	**70**.**1**	*—*	317.3	*—*	1.9	216.0	0.2	3
32	56	M	*—*	2.3	2.0	0.20	63.0	**1**.**4**	195.0	12.2	2.6	**46**.**0**	0.1	3
33	59	M	*—*	3.5	2.5	0.28	56.8	2.5	331.0	14.2	1.2	90.9	0.1	0
34	59	M	*—*	3.1	8.6	0.29	31.6	3.0	331.1	12.7	4.1	119.0	0.1	4
35	61	M	*—*	6.1	7.5	**0**.**17**	27.5	**1**.**2**	215.5	**11**.**9**	2.8	95.0	0.1	3
36	62	M	*—*	3.0	5.3	0.30	54.5	*—*	290.0	12.7	0.7	—	—	0
37	67	M	*—*	7.1	11.1	**0**.**16**	**65**.**8**	3.1	232.9	14.0	1.2	159.0	1.7	2
38	67	M	*—*	4.8	5.2	**0**.**52**	28.9	**1**.**8**	350.0	14.0	0.6	**49**.**8**	0.3	3
39	67	M	*—*	4.7	14.0	**0**.**17**	38.0	3.4	260.0	12.3	1.0	**528**.**0**	0.4	3
40	67	M	*—*	4.1	4.1	**0**.**14**	55.3	2.7	361.0	**11**.**6**	0.8	102.0	0.1	1
41	68	M	*—*	4.5	5.5	0.30	23.2	2.3	263.8	12.4	**0**.**2**	96.2	0.1	4
42	68	M	*—*	4.8	3.4	0.28	30.6	**1**.**9**	347.6	13.1	0.8	99.6	0.1	4
43	68	M	*—*	11.2	11.8	0.20	29.4	2.6	331.0	12.6	0.7	310.0	0.1	2

Abbreviations: —, measurement not taken; F, women; M, men; ND, not detectable; post-F, postmenopausal women; pre-F, pre/perimenopausal women.

All reference ranges pertain to adults. Data points outside the reference range are in bold.

Reference ranges for premenopausal women include the ovulatory cycle at the follicular phase, periovulatory phase, and luteal phase.

^*a*^F denotes premenopausal women.

^*b*^Denotes samples excluded from the hormone analysis due to a hysterectomy, oophorectomy, or cancer.

## Discussion

Based on available fertility data (Table 1) and hormone profile analyses (Table 2) of *GNRHR* p.Q106R heterozygote and wild-type individuals, we report no differences between the 2 genotypes. In fact, there were no differences in median levels of the reproductive hormones (LH, FSH, estradiol, free testosterone, SHBG, and DHEA-SO_4_) between wild-type and heterozygous individuals (Table 2). The only difference in median hormone levels observed was a lower median TSH level in heterozygous men 50 years and older. However, 1 out of 19 men in this category falls outside the acceptable normal reference ranges for TSH (0.3–3 mIU/L), compared to 3 out of 469 wild-type men aged 50 and above. Questionnaire data from our heterozygous cohort shows that none of the individuals reported a thyroid disorder when specifically asked during the questionnaire interview. Low TSH levels coupled with normal free T4 and T3 levels are consistent with subclinical hyperthyroidism, and it is well documented that thyroid malfunction may disrupt menstrual patterns and ovulation disorders in women and may also cause infertility in both sexes [36, 37]. A study on individuals with polycystic ovary syndrome does suggest that there are pathophysiological links between the *GNRHR* locus and thyroid function [38]. However, more evidence than what the current literature supports is needed to show how GnRH neurons and thyroid hormones interact [39]. Furthermore, low TSH levels measured by immunoassays can also be due to interference by biotin [40, 41] or by endogenous antibodies [41-43].

Our findings indicate that the onset of puberty (in women) or the likelihood for an individual to bear offspring is not influenced by being heterozygous for *GNRHR* p.Q106R. This is corroborated by the high frequency of heterozygotes who had offspring, which is reflective of expected frequencies in a healthy population. In women, collection of data pertaining to the age at menarche was also particularly important since constitutional delay of growth and puberty and IHH form part of the same GnRH deficiency spectrum with shared pathogenic mechanisms and similar clinical phenotypes caused by variants at overlapping genetic loci [44]. Together with IHH, late menarche and constitutional delay of growth and puberty have been previously associated with homozygous *GNRHR* partial loss-of-function variants [15, 45]. Mild IHH phenotypes such as secondary hypothalamic amenorrhea may manifest if extraneous stressors such as extreme weight variation, strenuous exercise, or psychological stress become present in tandem with defects in the biology of GnRH attributed to monoallelic *GNRHR* variants [46-48]. This gene/environment interaction has also been observed for *FGFR1*, *ANOS1,* and *PROKR2* [48, 49]. In a cohort of GnRH-deficient patients (n = 397), Sykiotis et al identified Caucasian individuals harboring monoallelic autosomal recessive *GNRHR* mutations in 6% of the patient cohort, suggesting that when such variants in heterozygosity form oligogenic interactions due to endogamy or chance, inhibition of the hypothalamic-pituitary-gonadal axis occurs leading to IHH phenotype manifestations [49].

In a Maltese newborn cord-blood collection, we found the *GNRHR* p.Q106R variant (MAF = 0.029; n = 493) to be 10 times more frequent than that of the global population (MAF = 0.003; n = 282 638) and 6 times more frequent than the southern European population (MAF = 0.005, n = 11 596 [32]). We suspect that the high *GNRHR* p.Q106R carrier frequency in the Maltese population is due to a founder effect and contributes to a high prevalence of autosomal recessive IHH locally. This variant has already been described to have founder attributes for heterozygote carriers in other populations (European, North and South American, and South Asian) [19, 50].

While pathogenic variants are gradually eliminated from the human gene pool, founder variants tend to persist and are passed down through the generations [19]. However, counterintuitive to natural selection, this persistence stems from heterozygotes being relatively protected against certain diseases with a selective advantage over their homozygote alternative and wild-type counterparts. It has been proposed that variants like *GNRHR* p.Q106R may play a role in impairing the reproductive function of a population during adverse temporal circumstances such as states of destitution, environmental calamities, climate change, and population migration that are disadvantageous and taxing on the energy-demanding needs of pregnancy and survival [51]. Reports of reversible functional hypothalamic amenorrhea in women with heterozygous variants in IHH-related genes [47, 48] support this hypothesis. This may have imparted evolutionary advantageous properties to women and their future offspring with flexibility and reversibility of the hypothalamic-pituitary-gonadal axis to resume GnRH function during more favorable conditions. For this reason, such founder variants remain conserved within the gene pool [52].

There are multiple reports of compound *GNRHR* heterozygous patients in whom IHH can be explained by the presence of 2 different *GNRHR* variant alleles [23, 53, 54]. However, the presence of the monoallelic *GNRHR* p.Q106R in multiple individuals from the MAMI cohort with normal hormone levels, puberty, and fertility reinforces the premise that additional deleterious variants in other genes must be present in IHH patients in whom only a single *GNRHR* variant allele is identified. These additional variants would adversely modulate GnRH signaling through digenic or oligogenic inheritance [49, 55, 56]. Depending on the number and the nature of other contributing genes and alleles that partake in this mode of inheritance, one may expect variable degrees of expressivity in the reproductive and pathophysiological phenotypes of the condition. This does not exclude potential undiscovered genes from interacting pleiotropically with *GNRHR* [49, 57, 58].

In this study we show that *GNRHR* p.Q106R heterozygotes do not have fertility issues or impaired gonadotropin and sex steroid hormone levels. Thus, *GNRHR* heterozygotes who exhibit IHH characteristics must have at least 1 other variant in a different IHH causative gene. Clinically this is an important consideration, particularly for diagnostic laboratories making use of gene panels, since the IHH phenotype of individuals with monogenic *GNRHR* variants cannot be explained solely by this heterozygosity.

## Data Availability

Some data sets generated during and/or analysed during the current study are not publicly available but are available from the corresponding author upon reasonable request.

## References

[1] Stamou MI, Cox KH, Crowley WF, Jr. Discovering genes essential to the hypothalamic regulation of human reproduction using a human disease model: adjusting to life in the “-omics” era. Endocr Rev. 2016;2016(1):4‐22. 27454361 10.1210/er.2015-1045.2016.1 PMC6958992

[2] Topaloglu AK . Update on the genetics of idiopathic hypogonadotropic hypogonadism. J Clin Res Pediatr Endocrinol. 2017;9(Suppl 2):113‐122. 29280744 10.4274/jcrpe.2017.S010 PMC5790323

[3] Amato LGL, Montenegro LR, Lerario AM, et al New genetic findings in a large cohort of congenital hypogonadotropic hypogonadism. Eur J Endocrinol. 2019;181(2):103‐119. 31200363 10.1530/EJE-18-0764

[4] Kim JH, Seo GH, Kim GH, et al Targeted gene panel sequencing for molecular diagnosis of kallmann syndrome and normosmic idiopathic hypogonadotropic hypogonadism. Exp Clin Endocrinol Diabetes. 2019;127(8):538‐544. 30216942 10.1055/a-0681-6608

[5] Stojilkovic SS, Bjelobaba I, Zemkova H. Ion channels of pituitary gonadotrophs and their roles in signaling and secretion. Front Endocrinol (Lausanne). 2017;8:126. 28649232 10.3389/fendo.2017.00126 PMC5465261

[6] Seeburg PH, Adelman JP. Characterization of cDNA for precursor of human luteinizing hormone releasing hormone. Nature. 1984;311(5987):666‐668. 6090951 10.1038/311666a0

[7] Duran-Pasten ML, Fiordelisio T. GnRH-Induced Ca(2+) signaling patterns and gonadotropin secretion in pituitary gonadotrophs. Functional adaptations to both ordinary and extraordinary physiological demands. Front Endocrinol (Lausanne). 2013;4:127. 24137156 10.3389/fendo.2013.00127 PMC3786263

[8] Flanagan CA, Manilall A. Gonadotropin-releasing hormone (GnRH) receptor structure and GnRH binding. Front Endocrinol (Lausanne). 2017;8:274. 29123501 10.3389/fendo.2017.00274 PMC5662886

[9] Bedecarrats GY, Kaiser UB. Mutations in the human gonadotropin-releasing hormone receptor: insights into receptor biology and function. Semin Reprod Med. 2007;25(5):368‐378. 17710733 10.1055/s-2007-984743

[10] Chevrier L, Guimiot F, de Roux N. GnRH receptor mutations in isolated gonadotropic deficiency. Mol Cell Endocrinol. 2011;346(1-2):21‐28. 21645587 10.1016/j.mce.2011.04.018

[11] Conn PM, Ulloa-Aguirre A. Trafficking of G-protein-coupled receptors to the plasma membrane: insights for pharmacoperone drugs. Trends Endocrinol Metab. 2010;21(3):190‐197. 20005736 10.1016/j.tem.2009.11.003 PMC2831145

[12] de Roux N . GnRH receptor and GPR54 inactivation in isolated gonadotropic deficiency. Best Pract Res Clin Endocrinol Metab. 2006;20(4):515‐528. 17161329 10.1016/j.beem.2006.10.005

[13] de Roux N, Young J, Misrahi M, et al A family with hypogonadotropic hypogonadism and mutations in the gonadotropin-releasing hormone receptor. N Engl J Med. 1997;337(22):1597‐1602. 9371856 10.1056/NEJM199711273372205

[14] Layman LC, Cohen DP, Jin M, et al Mutations in gonadotropin-releasing hormone receptor gene cause hypogonadotropic hypogonadism. Nat Genet. 1998;18(1):14‐15. 9425890 10.1038/ng0198-14

[15] Gianetti E, Hall JE, Au MG, et al When genetic load does not correlate with phenotypic spectrum: lessons from the GnRH receptor (GNRHR). J Clin Endocrinol Metab. 2012;97(9):E1798‐E1807. 22745237 10.1210/jc.2012-1264 PMC3431570

[16] Bianco SD, Kaiser UB. The genetic and molecular basis of idiopathic hypogonadotropic hypogonadism. Nat Rev Endocrinol. 2009;5(10):569‐576. 19707180 10.1038/nrendo.2009.177 PMC2864719

[17] Leanos-Miranda A, Ulloa-Aguirre A, Janovick JA, Conn PM. In vitro coexpression and pharmacological rescue of mutant gonadotropin-releasing hormone receptors causing hypogonadotropic hypogonadism in humans expressing compound heterozygous alleles. J Clin Endocrinol Metab. 2005;90(5):3001‐3008. 15728205 10.1210/jc.2004-2071

[18] Knollman PE, Janovick JA, Brothers SP, Conn PM. Parallel regulation of membrane trafficking and dominant-negative effects by misrouted gonadotropin-releasing hormone receptor mutants. J Biol Chem. 2005;280(26):24506‐24514. 15886197 10.1074/jbc.M501978200

[19] Choi JH, Balasubramanian R, Lee PH, et al Expanding the spectrum of founder mutations causing isolated gonadotropin-releasing hormone deficiency. J Clin Endocrinol Metab. 2015;100(10):E1378‐E1385. 26207952 10.1210/jc.2015-2262 PMC4596034

[20] Bhagavath B, Ozata M, Ozdemir IC, et al The prevalence of gonadotropin-releasing hormone receptor mutations in a large cohort of patients with hypogonadotropic hypogonadism. Fertil Steril. 2005;84(4):951‐957. 16213849 10.1016/j.fertnstert.2005.04.029

[21] Jardon-Valadez E, Ulloa-Aguirre A, Pineiro A. Modeling and molecular dynamics simulation of the human gonadotropin-releasing hormone receptor in a lipid bilayer. J Phys Chem B. 2008;112(34):10704‐10713. 18680336 10.1021/jp800544x

[22] Ulloa-Aguirre A, Janovick JA, Leanos-Miranda A, Conn PM. Misrouted cell surface GnRH receptors as a disease aetiology for congenital isolated hypogonadotrophic hypogonadism. Hum Reprod Update. 2004;10(2):177‐192. 15073146 10.1093/humupd/dmh015

[23] Kottler ML, Chauvin S, Lahlou N, et al A new compound heterozygous mutation of the gonadotropin-releasing hormone receptor (L314X, Q106R) in a woman with complete hypogonadotropic hypogonadism: chronic estrogen administration amplifies the gonadotropin defect. J Clin Endocrinol Metab. 2000;85(9):3002‐3008. 10999776 10.1210/jcem.85.9.6783

[24] Bedecarrats GY, Linher KD, Janovick JA, et al Four naturally occurring mutations in the human GnRH receptor affect ligand binding and receptor function. Mol Cell Endocrinol. 2003;205(1-2):51‐64. 12890567 10.1016/s0303-7207(03)00201-6

[25] Bedecarrats GY, Linher KD, Kaiser UB. Two common naturally occurring mutations in the human gonadotropin-releasing hormone (GnRH) receptor have differential effects on gonadotropin gene expression and on GnRH-mediated signal transduction. J Clin Endocrinol Metab. 2003;88(2):834‐843. 12574221 10.1210/jc.2002-020806

[26] Boehm U, Bouloux PM, Dattani MT, et al Expert consensus document: European consensus statement on congenital hypogonadotropic hypogonadism–pathogenesis, diagnosis and treatment. Nat Rev Endocrinol. 2015;11(9):547‐564. 26194704 10.1038/nrendo.2015.112

[27] Attard R, Dingli P, Doggen CJM, Cassar K, Farrugia R, Wettinger SB. The impact of passive and active smoking on inflammation, lipid profile and the risk of myocardial infarction. Open Heart. 2017;4(2):e000620. 28878948 10.1136/openhrt-2017-000620 PMC5574419

[28] Miller SA, Dykes DD, Polesky HF. A simple salting out procedure for extracting DNA from human nucleated cells. Nucleic Acids Res. 1988;16(3):1215. 3344216 10.1093/nar/16.3.1215 PMC334765

[29] Pleven A . The use of High Throughput Sequencing to Identify Mutations Contributing to Idiopathic Hypogonadotropic Hypogonadism. University of Malta; 2017.

[30] He C, Holme J, Anthony J. SNP genotyping: the KASP assay. Methods Mol Biol. 2014;1145:75‐86. 24816661 10.1007/978-1-4939-0446-4_7

[31] Ho CK, Stoddart M, Walton M, Anderson RA, Beckett GJ. Calculated free testosterone in men: comparison of four equations and with free androgen index. Ann Clin Biochem. 2006;43(Pt 5):389‐397. 17036414 10.1258/000456306778520115

[32] Karczewski KJ, Francioli LC, Tiao G, et al The mutational constraint spectrum quantified from variation in 141,456 humans. Nature. 2020;581(7809):434‐443. 32461654 10.1038/s41586-020-2308-7 PMC7334197

[33] Baulieu EE, Thomas G, Legrain S, et al Dehydroepiandrosterone (DHEA), DHEA sulfate, and aging: contribution of the DHEAge study to a sociobiomedical issue. Proc Natl Acad Sci U S A. 2000;97(8):4279‐4284. 10760294 10.1073/pnas.97.8.4279 PMC18228

[34] Elmlinger MW, Kuhnel W, Wormstall H, Doller PC. Reference intervals for testosterone, androstenedione and SHBG levels in healthy females and males from birth until old age. Clin Lab. 2005;51(11-12):625‐632. 16329620

[35] Henderson VW . Aging, estrogens, and episodic memory in women. Cogn Behav Neurol. 2009;22(4):205‐214. 19996872 10.1097/WNN.0b013e3181a74ce7 PMC2791907

[36] Yang J, Zhou X, Zhang X, et al Analysis of the correlation between lipotoxicity and pituitary-thyroid axis hormone levels in men and male rats. Oncotarget. 2016;7(26):39332‐39344. 27322428 10.18632/oncotarget.10045 PMC5129936

[37] Coelho Neto MA, Martins WP, Melo AS, Ferriani RA, Navarro PA. Subclinical hypothyroidism and intracytoplasmic sperm injection outcomes. Rev Bras Ginecol Obstet. 2016;38(11):552‐558. 27852097 10.1055/s-0036-1593986 PMC10309385

[38] Li Q, Yang G, Wang Y, et al Common genetic variation in the 3'-untranslated region of gonadotropin-releasing hormone receptor regulates gene expression in cella and is associated with thyroid function, insulin secretion as well as insulin sensitivity in polycystic ovary syndrome patients. Hum Genet. 2011;129(5):553‐561. 21274726 10.1007/s00439-011-0954-4

[39] Sun J, Hui C, Xia T, et al Effect of hypothyroidism on the hypothalamic-pituitary-ovarian axis and reproductive function of pregnant rats. BMC Endocr Disord. 2018;18(1):30. 29793475 10.1186/s12902-018-0258-y PMC5968710

[40] Ylli D, Soldin SJ, Stolze B, et al Biotin interference in assays for thyroid hormones, thyrotropin and thyroglobulin. Thyroid. 2021;31(8):1160‐1170. 34042535 10.1089/thy.2020.0866 PMC8420951

[41] Ghazal K, Brabant S, Prie D, Piketty ML. Hormone immunoassay interference: a 2021 update. Ann Lab Med. 2022;42(1):3‐23. 34374345 10.3343/alm.2022.42.1.3 PMC8368230

[42] Dahll LK, Haave EM, Dahl SR, Aas FE, Thorsby PM. Endogenous anti-streptavidin antibodies causing erroneous laboratory results more common than anticipated. Scand J Clin Lab Invest. 2021;81(2):92‐103. 33502256 10.1080/00365513.2020.1858493

[43] Berth M, Willaert S, De Ridder C. Anti-streptavidin IgG antibody interference in anti-cyclic citrullinated peptide (CCP) IgG antibody assays is a rare but important cause of false-positive anti-CCP results. Clin Chem Lab Med. 2018;56(8):1263‐1268. 29466233 10.1515/cclm-2017-1153

[44] Zhu J, Choa RE, Guo MH, et al A shared genetic basis for self-limited delayed puberty and idiopathic hypogonadotropic hypogonadism. J Clin Endocrinol Metab. 2015;100(4):E646‐E654. 25636053 10.1210/jc.2015-1080 PMC4399304

[45] Howard SR . The genetic basis of delayed puberty. Front Endocrinol (Lausanne). 2019;10:423. 31293522 10.3389/fendo.2019.00423 PMC6606719

[46] Fourman LT, Fazeli PK. Neuroendocrine causes of amenorrhea–an update. J Clin Endocrinol Metab. 2015;100(3):812‐824. 25581597 10.1210/jc.2014-3344 PMC4333037

[47] Caronia LM, Martin C, Welt CK, et al A genetic basis for functional hypothalamic amenorrhea. N Engl J Med. 2011;364(3):215‐225. 21247312 10.1056/NEJMoa0911064 PMC3045842

[48] Delaney A, Burkholder AB, Lavender CA, et al Increased burden of rare sequence variants in GnRH-associated genes in women with hypothalamic amenorrhea. J Clin Endocrinol Metab. 2020;106(3):e1441‐e1452. 10.1210/clinem/dgaa609 PMC7947783 32870266

[49] Sykiotis GP, Plummer L, Hughes VA, et al Oligogenic basis of isolated gonadotropin-releasing hormone deficiency. Proc Natl Acad Sci U S A. 2010;107(34):15140‐15144. 20696889 10.1073/pnas.1009622107 PMC2930591

[50] Caburet S, Fruchter RB, Legois B, Fellous M, Shalev S, Veitia RA. A homozygous mutation of GNRHR in a familial case diagnosed with polycystic ovary syndrome. Eur J Endocrinol. 2017;176(5):K9‐K14. 28348023 10.1530/EJE-16-0968

[51] Avbelj Stefanija M, Jeanpierre M, Sykiotis GP, et al An ancient founder mutation in PROKR2 impairs human reproduction. Hum Mol Genet. 2012;21(19):4314‐4324. 22773735 10.1093/hmg/dds264 PMC3441126

[52] Cioppi F, Riera-Escamilla A, Manilall A, et al Genetics of ncHH: from a peculiar inheritance of a novel GNRHR mutation to a comprehensive review of the literature. Andrology. 2019;7(1):88‐101. 30575316 10.1111/andr.12563

[53] Karges B, Karges W, Mine M, et al Mutation ala(171)Thr stabilizes the gonadotropin-releasing hormone receptor in its inactive conformation, causing familial hypogonadotropic hypogonadism. J Clin Endocrinol Metab. 2003;88(4):1873‐1879. 12679486 10.1210/jc.2002-020005

[54] Seminara SB, Beranova M, Oliveira LM, Martin KA, Crowley WF, Jr., Hall JE. Successful use of pulsatile gonadotropin-releasing hormone (GnRH) for ovulation induction and pregnancy in a patient with GnRH receptor mutations. J Clin Endocrinol Metab. 2000;85(2):556‐562. 10690855 10.1210/jcem.85.2.6357

[55] Goncalves CI, Aragues JM, Bastos M, et al GNRHR biallelic and digenic mutations in patients with normosmic congenital hypogonadotropic hypogonadism. Endocr Connect. 2017;6(6):360‐366. 28611058 10.1530/EC-17-0104 PMC5527354

[56] Cerrato F, Shagoury J, Kralickova M, et al Coding sequence analysis of GNRHR and GPR54 in patients with congenital and adult-onset forms of hypogonadotropic hypogonadism. Eur J Endocrinol. 2006;155(Suppl 1):S3‐S10. 17074994 10.1530/eje.1.02235

[57] Sarfati J, Guiochon-Mantel A, Rondard P, et al A comparative phenotypic study of kallmann syndrome patients carrying monoallelic and biallelic mutations in the prokineticin 2 or prokineticin receptor 2 genes. J Clin Endocrinol Metab. 2010;95(2):659‐669. 20022991 10.1210/jc.2009-0843

[58] Maggi R, Cariboni AM, Marelli MM, et al GnRH and GnRH receptors in the pathophysiology of the human female reproductive system. Hum Reprod Update. 2016;22(3):358‐381. 26715597 10.1093/humupd/dmv059

